# Functional analysis of thermo-sensitive TRPV1 in an aquatic vertebrate, masu salmon (*Oncorhynchus masou ishikawae*)

**DOI:** 10.1016/j.bbrep.2022.101315

**Published:** 2022-07-21

**Authors:** A. Yoshimura, S. Saito, C.T. Saito, K. Takahashi, M. Tominaga, T. Ohta

**Affiliations:** a Department of Veterinary Pharmacology, Tottori University, Tottori, Japan; b Division of Cell Signaling, National Institute for Physiological Sciences, National Institutes of Natural Sciences, Okazaki, Aichi, 444-8787, Japan; c Thermal Biology Group, Exploratory Research Center on Life and Living Systems (ExCELLS), National Institute of Natural Sciences, Aichi, Japan; d Joint Graduate School of Veterinary Sciences, Gifu University, Tottori University, Tottori, Japan

**Keywords:** Capsaicin, Salmonids, Species differences, Thermo-sensor, HEPES, N-2-hydroxyethyl-piperazine-2-ethanonesufonic acid, Om, *Oncorhynchus masou ishikawae*, TRPV1, Transient receptor potential vanilloid 1

## Abstract

Transient receptor potential vanilloid 1 (TRPV1) is mainly expressed in nociceptive primary sensory neurons and acts as a sensor for heat and capsaicin. The functional properties of TRPV1 have been reported to vary among species and, in some cases, the species difference in its thermal sensitivity is likely to be associated with thermal habitat conditions. To clarify the functional properties and physiological roles of TRPV1 in aquatic vertebrates, we examined the temperature and chemical sensitivities of TRPV1 in masu salmon (*Oncorhynchus masou ishikawae*, Om) belonging to a family of salmonids that generally prefer cool environments. First, behavioral experiments were conducted using a video tracking system. Application of capsaicin, a TRPV1 agonist, induced locomotor activities in juvenile Om. Increasing the ambient temperature also elicited locomotor activity potentiated by capsaicin. RT-PCR revealed TRPV1 expression in gills as well as spinal cord. Next, electrophysiological analyses of OmTRPV1 were performed using a two-electrode voltage-clamp technique with a *Xenopus* oocyte expression system. Heat stimulation evoked an inward current in heterologously expressed OmTRPV1. In addition, capsaicin produced current responses in OmTRPV1-expressing oocytes, but higher concentrations were needed for its activation compared to the mammalian orthologues. These results indicate that Om senses environmental stimuli (heat and capsaicin) through the activation of TRPV1, and this channel may play important roles in avoiding environments disadvantageous for survival in aquatic vertebrates.

## Highlights

• Capsaicin evoked behavioral responses of *Oncorhynchus masou ishikawae* (Om).
• The behavioral response to heat was potentiated by capsaicin.
• Heterologously expressed OmTRPV1 was activated by heat and capsaicin.

## 1 Introduction

Ambient temperature is one of the most critical environmental factors affecting a variety of physiological functions influencing homeostasis in organisms. Thus, temperature sensation is necessary for both homeotherms and ectotherms to adapt to changing environmental conditions. Noxious heat sensation plays important roles for survival since it is required for the avoidance of harmful thermal environments. In vertebrates, peripheral sensory neurons relay temperature information to the central nervous system. During the initiation of signal transduction, thermal stimuli are transduced into electrical signals and a subset of ion channels that are thermally activated plays crucial roles in this process [1]. Several temperature-sensitive ion channels belong to the transient receptor potential (TRP) ion channel superfamily and are called “thermoTRPs” [2]. They are multimodal receptors that are activated by temperature as well as other physical and chemical stimuli [3]. ThermoTRPs have been characterized in diverse animal species over the past two decades, which has illuminated their functional diversification in the course of evolution [4,5].

Among thermoTRP channels, TRP vanilloid 1 (TRPV1) is well conserved among vertebrate species and serves as a sensor for noxious stimuli. Mammalian TRPV1 is activated by noxious heat and capsaicin. The temperature threshold of activation for human and rodent TRPV1 is 42 °C or more [6]. We previously reported that TRPV1 from several clawed frogs belonging to genus *Xenopus* was activated by heat higher than approximately 40 °C [7] and clawed frogs showed nocifensive behavior close to their thermal activation thresholds of TRPV1 [8]. Therefore, the thermal sensitivity of TRPV1 in *Xenopus* species is similar to that of mammalian TRPV1, although *Xenopus* are ectothermic and fully aquatic species. TRPV1 was previously cloned from zebrafish and its functional properties were characterize using a heterologous expression system. Zebrafish TRPV1 was reported to be activated by temperatures higher than 25 °C, which was lower than the optimal rearing temperature for this species (28.5 °C), although TRPV1 was shown to be involved in noxious heat detection by using knockdown zebrafish embryos [9]. Currently, functional characterizations of TRPV1 have not been performed using fish except for zebrafish. Thus, the physiological role of TRPV1 in detecting environmental temperatures has remained to be elucidated, especially in fish species.

In this study, to reveal the functional significance of TRPV1 in aquatic vertebrates, we examined behavioral changes of masu salmon (*Oncorhynchus masou ishikawae,* Om), an anadromous fish that prefers low temperatures, in response to thermal and chemical stimuli. In addition, we cloned OmTRPV1 and observed current responses to heat and capsaicin by heterologously expressing it in *Xenopus* oocytes.

## 2 Materials and methods

### 2.1 Experimental animals

Om (10.3 ± 1.2 cm, 14.4 ± 5.6 g) were purchased from Hinakura fish farm ( Okayama, Japan) and maintained in a water tank with air circulated (kept under 10 °C), fed once every 3 days. The river water was drawn from this fish farm and stored in a refrigerator. This river water was used for fish rearing and the behavioral experiments. For electrophysiological experiments, mature *Xenopus laevis* females were purchased from Hamamatsu Seibutsu Kyouzai (Shizuoka, Japan) and reared at approximately 18 °C on a 14h:10h light/dark cycle. All procedures involving the care and use of *X. laevis* were approved by the Animal Experimentation Committee of the National Institute for Physiological Sciences (Japan).

### 2.2 Behavioral experiment

**Fig. 1:**
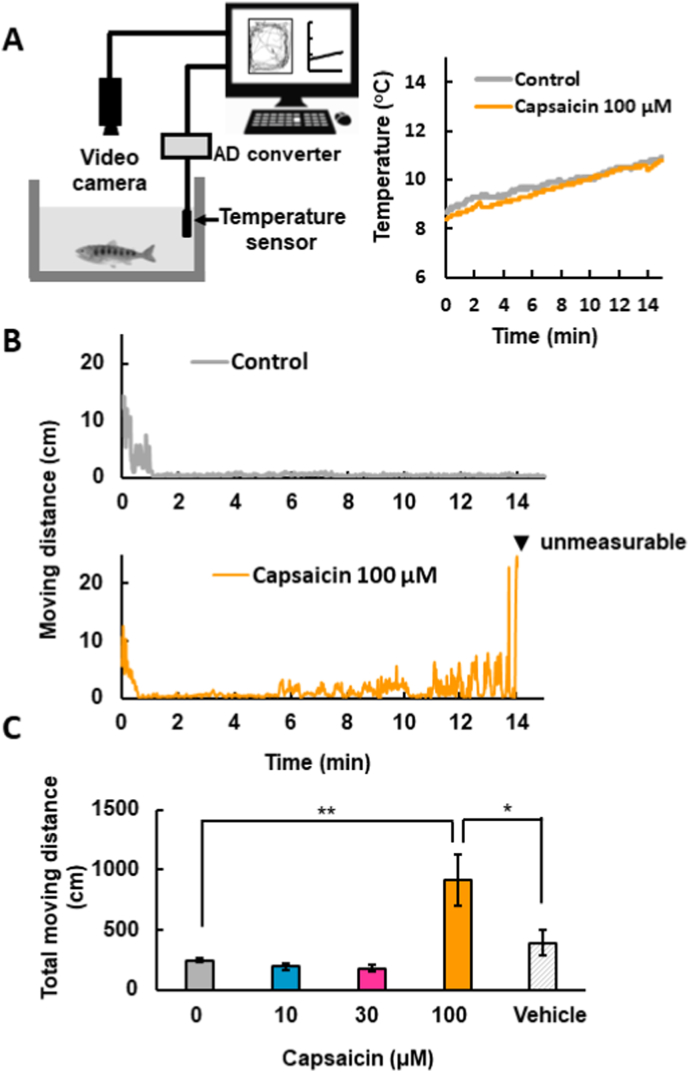
Behavioral responses to capsaicin in *Oncorhynchus masou ishikawae*. (A) Left: Diagram of the behavioral experiment setting. Each *Oncorhynchus masou ishikawae* (Om) was released into a plastic case filled with water in the presence or absence of capsaicin, and the locomotor activity of Om was monitored using a video camera. At the same time, water temperature was monitored by using a temperature sensor. These data were stored on the hard disk of a PC in conjunction with an AD converter. Right: Changes in water temperature for 15 min in the water case during the analysis. Non-treated water was used as a control (gray line). Capsaicin was added to water at 100 μM (red line). (B) Typical changes in the moving distance of Om in non-treated water (upper: control, gray line) and capsaicin (100 μM)-added water (lower: red line). Moving distances were plotted every 1 s for 15 min. The arrowhead (▼) indicates the unmeasurable point, at which data could not be collected because the fish moved erratically. (C) Summarized effects of capsaicin on the total moving distance for 15 min. Columns with vertical lines show mean ± SEM (control [water alone]: n = 7, capsaicin 10 μM: n = 7, 30 μM: n = 7, 100 μM: n = 7, vehicle, ethanol 0.1%, equivalent to 100 μM capsaicin: n = 5). *, p < 0.05, **, p < 0.01 by one-way ANOVA with the Tukey-Kramer test. (For interpretation of the references to colour in this figure legend, the reader is referred to the Web version of this article.)

The locomotor activity of Om was assessed using a video tracking system (Smart3.0, Bioresearch Center, Co., Ltd. Tokyo, Japan). Each Om was placed in a plastic case (12 cm in length, 8 cm in width, 15 cm in height) filled with river water (500 mL, 10 °C), and the locomotion was measured for 15 min. Water temperature was recorded by using a temperature probe (HT-1, Bioresearch Center) in conjunction with an AD converter (PowerLab, Bioresearch Center). To examine the effects of capsaicin, capsaicin dissolved in ethanol (0.1 M) as a stock solution was used. For control experiments, the same amount of ethanol was added as a vehicle. Heat stimulation was given by putting the plastic case with the Om on a thermo-bath (50 °C). Water temperature was gradually increased and the temperature at which the fish began to move was determined as the threshold temperature by the video tracking system while visual observations were also performed (Fig. 1). Erratic behaviors such as sideways overturning (overturn), upside-down inversion (inversion) and death were also observed visually and temperatures at which behavioral changes occurred were recorded. In each experiment, animals were used only once. They were quickly decapitated at the end of the experiment.

### 2.3 Molecular cloning

Total RNA was extracted from the excised Om spinal cord and used as template for RT-PCR with the following primers: OmTRPV1-F (5′-AGCATCCCTCCCTACTACTCAC-3′) and OmTRPV1-R (5′-AGGAGGATGCGAGAGCAGAC-3′). The RT-PCR product was further used as a template for the second round of PCR with the following primers: OmTRPV1-F-*Eco*RI (5′-ACTGAATTCATGAGCAAGTCAAAGGGCCCAGAG-3′) and OmTRPV1-R-*Not*I (5′-TAAGCGGCCGCTCAGACATGGTTGAGAGGAGAC-3′). The amplified DNA fragment containing OmTRPV1 was cloned into the pOX(+) vector with *Eco*RI and *Not*I. The nucleotide sequences of cloned TRPV1 were verified by sequencing multiple clones. The cDNA sequence data have been deposited in DDBJ and GenBank™ under accession number LC704440.

### 2.4 RT-PCR

Total RNAs from gills and spinal cord were extracted by using the RNeasy Mini Kit (Qiagen, Bothell, WA) and genomic DNA was digested by DNase I (Qiagen). First strand cDNA was synthesized from oligo (dT) primed total RNA with ReverTra Ace reverse transcriptase (Toyobo, Tokyo, Japan). Real-time RT-PCR was performed with 3 steps amplification protocol using SsoAdvanced^TM^ Universal SYBR Green Supermix (Bio-Rad, Hercules, CA, USA) with CFX Connect Real-Time PCR Detection System (Bio-Rad). Relative quantification of the starting mRNA copy numbers in gills and spinal cord using multiple replicates for each reaction was performed according to the ΔCt method. β-Actin was used as an endogenous reference gene.

For RT-PCR, cDNAs synthesized were subjected to PCR amplification with the use of Taq DNA polymerase (Promega, Tokyo, Japan) with a standard procedure using a T100™ thermal cycler (Bio-Rad ). We prepared cDNA samples with and without reverse transcriptase. The PCR products were resolved on 1% agarose gels and visualized by ethidium bromide staining followed by UV transillumination. The nucleotide sequences and the length of the expected PCR product for each primer pair were as follows: OmTRPV1, 5′-CGGTGGTCACTTTGTTGATG-3' (sense), 5′- TGAGCAGCAGGATGTAGGTG-3' (antisense) for 273 bp, Omβ-actin (accession number AB111056), 5′-TCTACGAGGGCTACGCTCTG-3' (sense), 5′-GCACAGCTTCTCCTTGATGTC-3' (antisense) for 212bp.

### 2.5 Two-electrode voltage-clamp

OmTRPV1 was expressed in *X. laevis* oocytes, and ionic currents were recorded with a two-electrode voltage-clamp method described previously [10]. Fifty nanoliters of OmTRPV1 cRNA (50 ng/μl) was injected into defolliculated oocytes, and ionic currents were recorded at 3- or 4-days post-injection using an OC-725C amplifier (Warner Instrument) and digitized by Digidata 1440 (Molecular Devices, U.S.A.). Oocytes were voltage-clamped at −60 mV. Capsaicin was diluted in a bath solution, ND96 (96 mM NaCl, 2 mM KCl, 1 mM MgCl_2_, 1.8 mM CaCl_2_, 5 mM HEPES) and applied to oocytes by perfusion. Heated or cooled ND96 was perfused to apply thermal stimulation and temperature was monitored with a thermistor located just beside the oocytes using a TC-344B (Warner Instruments, U.S.A.). Apparent thermal activation thresholds for OmTRPV1 were determined using *X. laevis* oocytes from three independent preparations by generating Arrhenius plots using Clampfit 10.4 (Molecular Devices, U.S.A.) and Origin 9J (OriginLab, U.S.A.) software. The current responses of OmTRPV1 to capsaicin stimulation were measured at four days after cRNA injection from *X. laevis* oocytes obtained from one to three independent preparations. The average current amplitudes obtained by different concentrations of capsaicin were fitted with a sigmoidal function to obtain the EC_50_ value using Origin 9J.

### 2.6 Chemicals

Capsaicin was from Sigma-Aldrich. All other chemicals were from Wako Pura Chem. (Osaka, Japan).

### 2.7 Data analysis

The data were presented as mean ± SEM (n = number of observation). For comparison of two groups, data were analyzed using the unpaired Student's *t*-test. For multiple comparisons, one-way ANOVA with the Tukey-Kramer test or nonparametric Steel-Dwass multiple-comparison test was used. Differences with a p-value of less than 0.05 were considered significant.

## 3 Results

### 3.1 Behavioral responses to capsaicin in *Oncorhynchus masou ishikawae* (Om)

In rodents, the administration of capsaicin elicits nocifensive behaviors via the activation of TRPV1. It has been reported that TRPV1 exhibits species differences in sensitivity to capsaicin [11]. Zebrafish TRPV1 was reported to be insensitive to capsaicin [9]; however, the responsiveness of Om to capsaicin is unclear. To clarify the sensitivity to capsaicin, the locomotor activity was analyzed in juvenile Om. As shown in Fig. 1A, after a fish was released into the case filled with water at approximately 10 °C, the preferable habitat temperature of Om, moving distance was measured and analyzed by using a video-tracking system for 15 min. During the experimental period, the water temperature was increased only 2 °C (Fig. 1A). In the control experiments without capsaicin, the Om swam just after release into the case while it generally stopped moving approximately after 1 min. The application of capsaicin at 100 μM induced swimming of Om from approximately 6 min and this activity gradually increased with time (Fig. 1B). Finally, video tracking of the Om became impossible since it started to exhibit abnormal swimming behaviors (marked with a filled rectangle in Fig. 1B). Capsaicin at 100 μM, but not at 10 μM or 30 μM, significantly increased the total moving distance in comparison with non-treated water. The vehicle did not increase the moving distance of Om (Fig. 1C). These results suggest that capsaicin elicits locomotor activity of Om at the individual level.

### 3.2 Capsaicin potentiates behavioral responses to heat in *Oncorhynchus masou ishikawae* (Om)

**Fig. 2:**
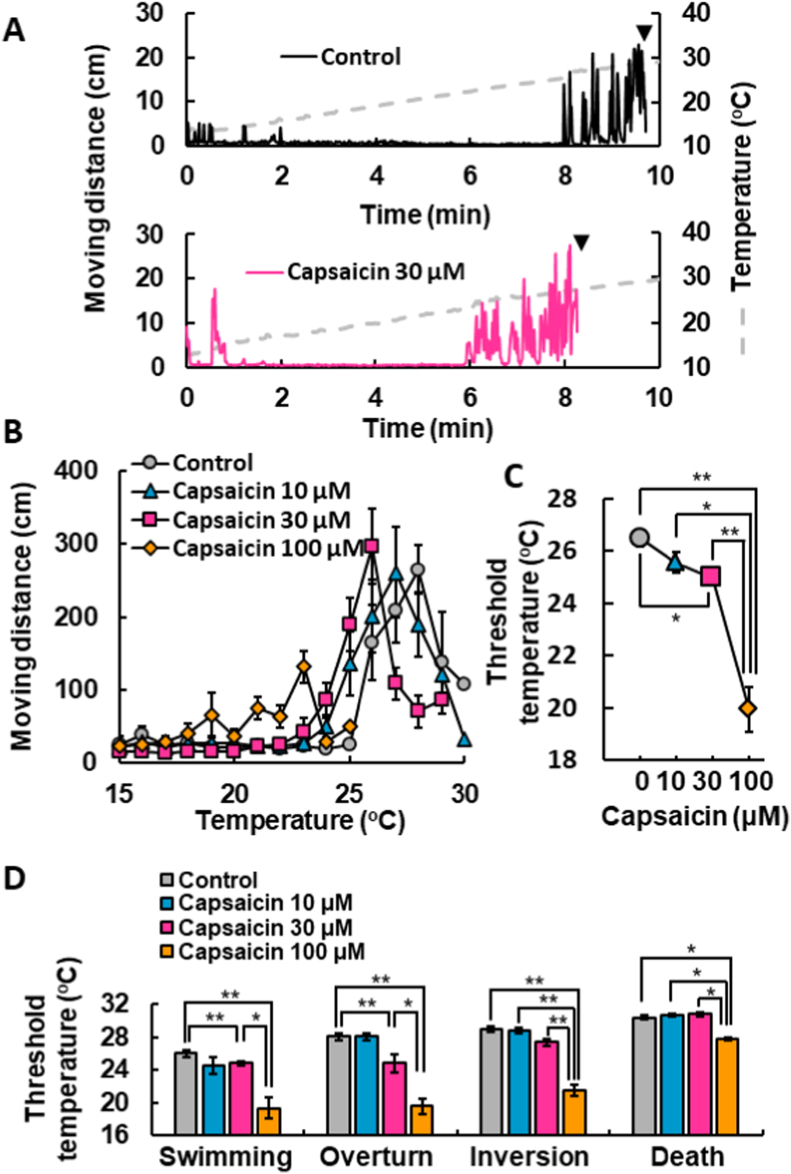
Potentiation of behavioral responses to heat by capsaicin in *Oncorhynchus masou ishikawae*. (A) Typical changes in the moving distance of Om with increasing water temperature (gray line) for 10 min in the absence (upper: control) or presence of 30 μM capsaicin (lower). The arrowhead (▼) shows the unmeasurable point as described in Fig. 1. Note that the onset of swimming induced by heat became earlier in the presence of capsaicin than in its absence. (B) Summarized changes in the moving distance caused by a heat ramp with various concentrations of capsaicin were plotted against temperature. Symbols with vertical lines show mean ± SEM (control [water alone]: n = 7, capsaicin 10 μM: n = 7, 30 μM: n = 7, 100 μM: n = 7). (C) Dose-dependent effects of capsaicin in the threshold temperatures for swimming in Om. Threshold temperatures were defined as temperatures when Om started to swim. Symbols with vertical lines show mean ± SEM. (D) Capsaicin decreases the threshold temperature eliciting behavioral changes. Increasing water temperature induced behavioral changes in the order of “start swimming”, disequilibrium such as “overturn” and “inversion”, and finally “death” of Om. Threshold temperatures were defined as temperatures at which a behavioral change occurred. Columns with vertical lines show mean ± SEM. These behavioral changes were determined by visual observations in addition to the software analysis shown in B. *, p < 0.05, **, p < 0.01 by nonparametric Steel-Dwass multiple-comparison test.

Next, we evaluated the thermal sensitivity of Om and/or the effect of capsaicin. In this experimental setting, water temperature was increased from 4 to 5 °C up to 30 °C. The temperature ramp induced swimming at 8 min, when the temperature of the water was around 25 °C (Fig. 2A). The addition of 30 μM capsaicin facilitated the initiation of swimming from approximately 6 min (at around 23 °C), indicating that threshold temperatures decreased compared to the non-treated control (Fig. 2A). It is worth noting that capsaicin at 30 μM was not effective to evoke locomotor activity at a constant temperature of around 10 °C (Fig. 1C). The analysis of thermal responses in the presence of various concentrations of capsaicin demonstrated that capsaicin significantly decreased the threshold temperatures for initiating swimming behaviors in a dose-dependent manner (Fig. 2B and C).

Increasing water temperature also induced erratic behaviors such as sideways overturning (overturn), upside-down inversion (inversion) and finally death in masu salmon. The average temperatures ±SEM of seven fish for these behavioral changes in the vehicle group were 26.5 ± 0.30 °C (start swimming), 28.5 ± 0.22 °C (overturn), 28.6 ± 0.17 °C (inversion) and 30.6 ± 0.28 °C (death). Again, capsaicin significantly decreased the temperature threshold for initiation of all behaviors compared to the vehicle control (Fig. 2D). These results suggest that Om is sensitive to heat, and capsaicin potentiates behavioral responses to heat at the individual level.

### 3.3 mRNA expression of TRPV1 in Om gills and spinal cord

**Fig. 3:**
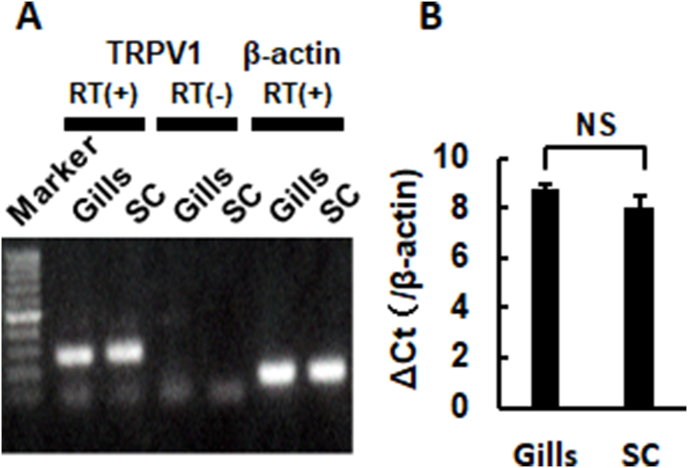
TRPV1 expression in the gills and spinal cord in Om. (A) mRNA expression of TRPV1 in gills and spinal cord (SC) of Om. RT-PCR was performed using cDNA obtained from each tissue with [RT(+)] or without [RT(−)] reverse transcriptase. RT-PCR was also carried out using the primer pair of β-actin as a control. (B) Summarized ΔCt values of TRPV1 mRNA in the gills and spinal cord by RT-qPCR analysis (n = 5). β-actin was used as an internal control. Columns with vertical lines show mean ± SEM. NS; Not significant by the unpaired Student's *t*-test (p = 0.130).

TRPV1 is mainly expressed in neural tissues in mammals, while TRPV1 is also expressed in gills in Atlantic Salmon [12]. Thus, we examined TRPV1 expression in the spinal cord and gills by RT-PCR and RT-qPCR. TRPV1 mRNA was detected from the cDNA extracted from Om gills as well as spinal cord (Fig. 3A), and the expression level of gills was not significantly different from that of spinal cord (Fig. 3B).

### 3.4 Activation of OmTRPV1 by heat and capsaicin stimulation

**Fig. 4:**
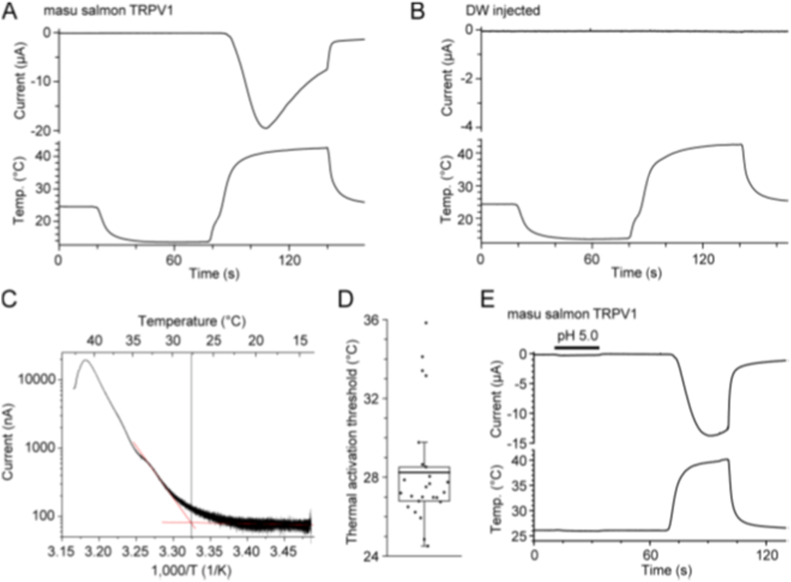
Heat sensitivity of OmTRPV1 expressed in *X. laevis* oocytes. (A, B) Representative current (upper) and temperature (lower) traces for heat stimulation in *Xenopus* oocytes injected with (A) OmTRPV1 cRNA and (B) distilled water (DW). (C) An Arrhenius plot of the current elicited by heat stimulation in A. (D) Summarized temperature thresholds for the activation by heat stimulation in OmTRPV1. The average temperature threshold for the activation, shown with a line, was 28.2 ± 0.6 °C (n = 25). The box indicates the upper and lower quartile range, and a whisker represents the highest or lowest non-outlier observations estimated using 1.5 times interquartile range. (E) A representative current trace for acid (pH 5.0) and heat stimulations in *Xenopus* oocytes injected with OmTRPV1 cRNA (n = 5).

Next, electrophysiological properties of OmTRPV1 were analyzed by using a *Xenopus* oocyte expression system. OmTRPV1 was heterologously expressed in *Xenopus* oocytes and ionic currents elicited by thermal stimulation were recorded. Since Om increased locomotor activity just above room temperature, we anticipated that Om would possess TRPV1 with low thermal activation thresholds for heat activation. The ionic current measurements were generally done at room temperature. Thus, the temperature was first decreased to approximately 15 °C, then heat stimulation was applied to *Xenopus* oocytes expressing OmTRPV1 to precisely examine its thermal responses. Heat stimulation evoked an inward current in *Xenopus* oocytes expressing OmTRPV1 (Fig. 4A). In contrast, control *X. laevis* oocytes injected with DW showed no response to thermal stimulation (Fig. 4B). The threshold temperature of heat activation for OmTRPV1 estimated via Arrhenius plot analysis (Fig. 4C) was 28.2 ± 0.6 °C (n = 25) (Fig. 4D). Since TRPV1 is activated by external acids in mammalian orthologue [13], the effect of acids on the OmTRPV1 was examined. As shown in Fig. 4E, however, the external acids (pH 5.0) did not activate OmTRPV1, but heat did.

**Fig. 5:**
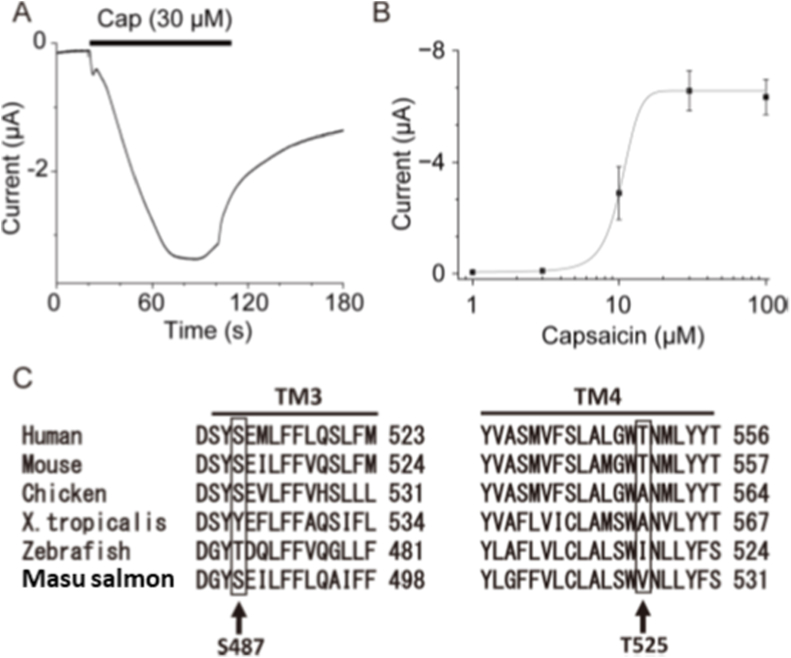
Capsaicin sensitivity of OmTRPV1 expressed in *Xenopus* oocytes. (A) A representative current trace for capsaicin (Cap, 30 μM) in *Xenopus* oocytes injected with OmTRPV1 cRNA. (B) A dose-dependent activation of OmTRPV1 to capsaicin stimulation. The current amplitudes were obtained from *Xenopus* oocytes at 4 days post OmTRPV1 cRNA injection, and datapoints were fitted with a sigmoidal function to obtain the EC_50_ value (10.4 μM). The each datapoint represents mean ± SEM (capsaicin 1 μM; n = 4, 3 μM; n = 5, 10 μM; n = 11, 30 μM; n = 11, 100 μM; n = 11). (C) Amino acid sequence alignments of vertebrate TRPV1s related to capsaicin sensitivity within transmembrane domain 3 (TM3) and TM4 are shown. Arrows indicate two amino acids associated with capsaicin sensitivity highlighted in boxes.

Next, the responses of OmTRPV1 to capsaicin were also examined. Application of capsaicin (30 μM) evoked an inward current in *Xenopus* oocytes expressing OmTRPV1 (Fig. 5A). The application of capsaicin elicited a dose-dependent response in OmTRPV1 (EC_50_ = 10.4 μM, Fig. 5B). Fig. 5C shows alignment of TRPV1 around the amino acids that interact with the capsaicin molecule. S487 and V525 in OmTRPV1 correspond to S512 and T550 in human and mouse TRPV1, respectively. Mutations in these two amino acids have been reported to reduce TRPV1 sensitivity to capsaicin [11,14]. In brief, our results showed that OmTRPV1 is functional to sense heat and capsaicin at the molecular level.

## 4 Discussion

Animals need to detect noxious heat, cold, and harmful chemicals for survival. In this study, to clarify the mechanism for sensing heat and chemicals in aquatic vertebrates, we investigated the behavioral responses to heat and capsaicin in Om in vivo and current responses to these stimuli in OmTRPV1-expressing *Xenopus* oocytes in vitro. We found that heat and capsaicin induced locomotor activity, and capsaicin potentiated the heat-induced behavioral responses in Om. In *Xenopus* oocytes expressing OmTRPV1, heat and capsaicin evoked membrane current responses.

In the behavioral experiment with Om, ramp heating induced swimming from 26 °C and erratic behaviors from 28 °C. These results indicated that Om might recognize temperatures above 26 °C as noxious. These observations fit well with thermal conditions of habitats occupied by Om since it is found in areas with water temperatures lower than 23 °C in natural streams [15]. In addition, capsaicin induced the locomotor activity of Om, and the concentration required for inducing behavioral responses was similar to that for *Xenopus tropicalis* [7,8]. These results suggest that OmTRPV1 is functional to detect environmental temperature and chemical stimuli.

The present data shows that individual Om are responsive to capsaicin. Since it has been reported that TRPV1 is expressed in gills of Atlantic salmons [12], we examined TRPV1 expression in Om gills by RT-PCR. These results revealed that TRPV1 expressed in gills, and the level of the expression was not significantly different from spinal cord. Therefore, these data indicated that at least the gills may act as capsaicin-sensitive organs in Om.

Capsaicin significantly decreased the threshold temperature initiating the swimming activity of Om. It was revealed that capsaicin potentiated the locomotor activity and the erratic behaviors evoked by increasing water temperatures. Similar potentiation effects of TRPV1 agonists on heat-induced responses have been reported. For example, protons potentiate the heat-evoked activity of TRPV1 by decreasing its thermal activation threshold in rat TRPV1 [13]. In addition, a reducing agent strongly increases thermally induced activity of TRPV1 in humans [16]. These previous findings support our idea that the potentiation effect of capsaicin on the heat-induced behaviors of Om seems to be dependent on TRPV1.

In the heterologous expression system, heat stimulation evoked an inward current in *Xenopus* oocytes expressing OmTRPV1 with an activation threshold temperature of ∼28 °C. The thermal activation threshold of OmTRPV1 was considerably lower than those of TRPV1 from mammals and clawed frogs, which are around 40 °C [7,8]. To our knowledge, this is the lowest thermal activation threshold for TRPV1 using the *Xenopus* oocyte expression system examined thus far. These findings were consistent with the fact that Om inhabit cool streams (<23 °C, [15]. However, the average thermal activation threshold of OmTRPV1 obtained using *Xenopus* oocytes (in *in vitro* experiments) was slightly higher than temperatures inducing locomotor activity of Om. This may have been due to the difference in the experimental methods for the detection of the heat-induced responses in vitro and in vivo. Furthermore, the cellular environment potentially altered the heat sensitivity of TRPV1. Indeed, thermal activation thresholds of TRPV1 varied among *Xenopus* oocytes as shown in Fig. 4D, and several *Xenopus* oocytes expressing OmTRPV1 showed thermal activation thresholds of around 26 °C. These observations may suggest that the thermal sensitivity of TRPV1 is affected by the cellular context, which could explain the discrepancy. Alternatively, the additional involvement of other thermosensitive molecules in Om cannot be ruled out because of the presence of various thermosensitive mechanisms [2]. Future investigation using TRPV1 knockout/knockdown salmon will clarify the role of TRPV1 in the heat-induced behaviors of Om.

Capsaicin evoked a current response in *Xenopus* oocytes expressing OmTRPV1. It has been reported that several amino acids are involved in the capsaicin sensitivity of TRPV1 such as serine 512 and threonine 550 in humans and mouse orthologues, located in the third and fourth putative transmembrane domains, respectively [11,14]. These two residues are known to be located in the binding pocket and form hydrogen bonds with the capsaicin molecule [17]. Comparison of the amino acid sequences of TRPV1 revealed that in OmTRPV1 only one amino acid changed to valine 525, corresponding to threonine 550 in the human and mouse (Fig. 5C). Instead, both critical amino acids are different in zebrafish TRPV1, which was reported to be insensitive to capsaicin [9]. This may be the reason for the species difference in capsaicin sensitivity between the two fish species since we previously reported the importance of both amino acids by mutagenesis analysis using *X. tropicalis* TRPV1, which exhibits reduced sensitivity, with major and minor effects on tyrosine 523 and alanine 561, respectively [8]. However, further mutagenesis analysis would be important to identify capsaicin-sensitive sites. In any case, TRPV1 has evolved to escape noxious heat and harmful chemicals species-specifically.

In conclusion, TRPV1 of Om is functional and plays an important role in the adaptation to fluctuating environmental temperatures. Thermal sensitivity of TRPV1 has altered in the course of evolution in order to fit with physiological and ecological traits in each species, resulting in functional diversification among species.

## Roles of authors

TO and SS designed the study; AY, SS, CS and TO conducted experiments; KT, CS, and MT conducted data analyses; AY, KT, SS and TO wrote the manuscript.

## Declaration of competing interest

We have no conflict-of-interest to declare.
